# The ZKIR Assay, a Real-Time PCR Method for the Detection of *Klebsiella pneumoniae* and Closely Related Species in Environmental Samples

**DOI:** 10.1128/AEM.02711-19

**Published:** 2020-03-18

**Authors:** Elodie Barbier, Carla Rodrigues, Geraldine Depret, Virginie Passet, Laurent Gal, Pascal Piveteau, Sylvain Brisse

**Affiliations:** a Agroécologie, AgroSup Dijon, INRAE, Université Bourgogne Franche-Comté, Dijon, France; b Institut Pasteur, Biodiversity and Epidemiology of Bacterial Pathogens, Paris, France; The Pennsylvania State University

**Keywords:** *Klebsiella*, phylogroup, soil, detection, screening, ZKIR qPCR, culture method, environment

## Abstract

Klebsiella pneumoniae is of growing public health concern due to the emergence of strains that are multidrug resistant, virulent, or both. Taxonomically, the K. pneumoniae complex (“Kp”) includes seven phylogroups, with Kp1 (K. pneumoniae *sensu stricto*) being medically prominent. Kp can be present in environmental sources such as soils and vegetation, which could act as reservoirs of animal and human infections. However, the current lack of screening methods to detect Kp in complex matrices limits research on Kp ecology. Here, we analyzed 1,001 genome sequences and found that existing molecular detection targets lack specificity for Kp. A novel real-time PCR method, the ZKIR (*zur-khe* intergenic region) assay, was developed and used to detect Kp in 96 environmental samples. The results were compared to a culture-based method using Simmons citrate agar with 1% inositol medium coupled to matrix-assisted laser desorption ionization–time of flight mass spectrometry identification. Whole-genome sequencing of environmental Kp was performed. The ZKIR assay was positive for the 48 tested Kp reference strains, whereas 88 non-Kp strains were negative. The limit of detection of Kp in spiked soil microcosms was 1.5 × 10^−1^ CFU g^−1^ after enrichment for 24 h in lysogeny broth supplemented with ampicillin, and it was 1.5 × 10^3^ to 1.5 × 10^4^ CFU g^−1^ directly after soil DNA extraction. The ZKIR assay was more sensitive than the culture method. Kp was detected in 43% of environmental samples. Genomic analysis of the isolates revealed a predominance of phylogroups Kp1 (65%) and Kp3 (32%), a high genetic diversity (23 multilocus sequence types), a quasi-absence of antibiotic resistance or virulence genes, and a high frequency (50%) of O-antigen type 3. This study shows that the ZKIR assay is an accurate, specific, and sensitive novel method to detect the presence of Kp in complex matrices and indicates that Kp isolates from environmental samples differ from clinical isolates.

**IMPORTANCE** The Klebsiella pneumoniae species complex Kp includes human and animal pathogens, some of which are emerging as hypervirulent and/or antibiotic-resistant strains. These pathogens are diverse and classified into seven phylogroups, which may differ in their reservoirs and epidemiology. Proper management of this public health hazard requires a better understanding of Kp ecology and routes of transmission to humans. So far, detection of these microorganisms in complex matrices such as food or the environment has been difficult due to a lack of accurate and sensitive methods. Here, we describe a novel method based on real-time PCR which enables detection of all Kp phylogroups with high sensitivity and specificity. We used this method to detect Kp isolates from environmental samples, and we show based on genomic sequencing that they differ in antimicrobial resistance and virulence gene content from human clinical Kp isolates. The ZKIR PCR assay will enable rapid screening of multiple samples for Kp presence and will thereby facilitate tracking the dispersal patterns of these pathogenic strains across environmental, food, animal and human sources.

## Abstract (precis)

The Klebsiella pneumoniae species complex Kp includes human and animal pathogens, some of which are emerging as hypervirulent and/or antibiotic-resistant strains. These pathogens are diverse and classified into seven phylogroups, which may differ in their reservoirs and epidemiology. Proper management of this public health hazard requires a better understanding of Kp ecology and routes of transmission to humans. So far, detection of these microorganisms in complex matrices such as food or the environment has been difficult due to a lack of accurate and sensitive methods. Here, we describe a novel method based on real-time PCR which enables detection of all Kp phylogroups with high sensitivity and specificity. We used this method to detect Kp isolates from environmental samples, and we show based on genomic sequencing that they differ in antimicrobial resistance and virulence gene content from human clinical Kp isolates. The ZKIR PCR assay will enable rapid screening of multiple samples for Kp presence and will thereby facilitate tracking the dispersal patterns of these pathogenic strains across environmental, food, animal and human sources.

## INTRODUCTION

Klebsiella pneumoniae is one of the leading causes of multidrug-resistant (MDR) health care-acquired infections, with increasing rates of resistance to carbapenems and other last resort antibiotics being reported (1, 2). Furthermore, is also an important agent of severe community-acquired infections (so-called “hypervirulent” strains) in healthy persons (3), with recent worrisome reports of convergence between hypervirulent and MDR phenotypes (1, 4). K. pneumoniae is recognized as a colonizer of the throat and the intestinal tract in humans and animals (5–7).

The main sources of human exposure to K. pneumoniae are not well defined. Previous studies highlighted the large distribution of K. pneumoniae in outdoor environments, including water, sewage, soil, and plants (8–13). Animal and human food, particularly retail meat or salad, may also be contaminated (6, 14, 15). Many studies suggest that food, water, and/or environmental exposure may be associated with virulent and/or antibiotic-resistant K. pneumoniae in humans (13, 14, 16, 17). However, little is known of the relative contributions of these different sources of transmission. Although such information is a prerequisite to control efficiently transmission routes and reduce exposure, the ecology of K. pneumoniae is currently poorly understood.

The systematics of K. pneumoniae has been refined through recent taxonomic updates, which highlighted the existence of seven phylogroups (phylogroup 1 [Kp1] to Kp7), corresponding to distinct taxa, within Klebsiella pneumoniae *sensu lato*. The K. pneumoniae species complex includes five different species: K. pneumoniae *sensu stricto* (Kp1), *K. quasipneumoniae* subsp. *quasipneumoniae* (Kp2) and subsp. *similipneumoniae* (Kp4), *K. variicola* subsp. *variicola* (Kp3) and subsp. *tropica* (Kp5), “*K. quasivariicola*” (Kp6), and *K. africana* (Kp7) (8, 18–20). Most of these taxa are still often misidentified as “K. pneumoniae” or “*K. variicola*” due to the unsuitability of traditional clinical microbiology methods to distinguish among members of the Kp complex (21). Henceforth, we use the “Kp” abbreviation to refer collectively to the seven phylogroups of the K. pneumoniae species complex and will reserve “K. pneumoniae” for K. pneumoniae *sensu stricto* (i.e., phylogroup Kp1). Since all Kp organisms are potentially pathogenic for humans and animals and can share acquired resistance and virulence genes, it is important that the seven taxa be considered together when investigating the routes of transmission and ecology of Kp.

Detection of Kp is not well integrated in food or environmental microbiological surveillance programs, and there is a general lack of tools and procedures for its detection and quantification. Culture-based laboratory methods used for the detection of microorganisms in complex matrices are time-consuming and have a low throughput. Moreover, Kp culture methods have not been validated so far for food safety screening. Some molecular methods (without need of sequencing) have been proposed over the years for the rapid detection of Kp (22–26). These organisms target the 16S-23S rRNA internal transcribed spacer sequence (ITS) (22), coding sequences of *tyrB* (26), *khe* (25, 27), chromosomal beta-lactamase (*bla*) genes (28, 29), or other molecular targets (23). Some of these targets are described as able to detect the Kp complex (22), whereas others were designed for specific members of the Kp complex, such as Kp1 and Kp3 (23, 24).

Real-time PCR is a powerful approach for the rapid detection and quantification of microorganisms in complex matrices (30). This approach presents multiple advantages, including easy standardization and high throughput. The aims of this work were (i) to define the phylogenetic distribution of previously proposed molecular targets for Kp detection, in light of recent taxonomic updates, and (ii) to develop a real-time PCR method for the rapid, specific and sensitive detection of all Kp members. (iii) In addition, we used a novel qPCR method to detect Kp in environmental samples and explored the genomic features (including antibiotic resistance and virulence genes) of the recovered Kp isolates.

## RESULTS

### Revisiting the phylogenetic distribution of proposed molecular targets for Kp detection.

**FIG 1:**
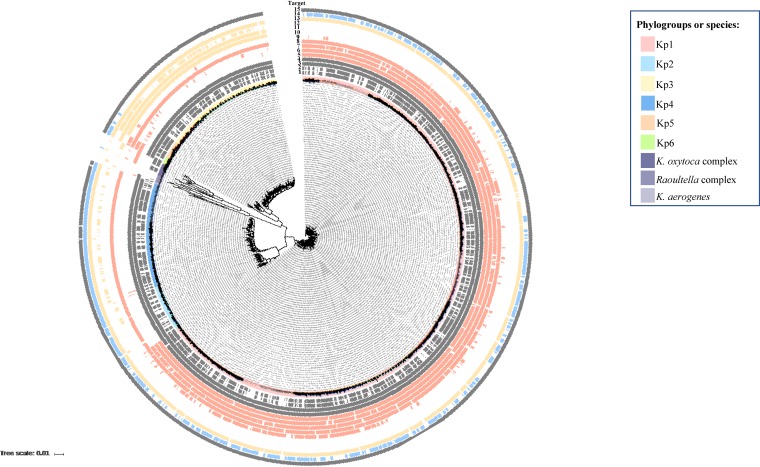
Phylogenetic distribution of the molecular targets for K. pneumoniae detection described in the literature. The ZKIR target sequence corresponds to target 15; other targets are given in Table 1. The inner circle colored sectors correspond to K. pneumoniae phylogroups or other *Klebsiella* species (see color key). Molecular targets were detected in the corresponding genomes using BLASTN with 80% nucleotide identity and 80% length coverage.

Fourteen molecular targets were found in the literature (Fig. 1; Table 1 ) (22–28, 31). Four of them were proposed to detect the Kp complex, while others were designed for specific members of this complex (K. pneumoniae, *K. variicola*, or *K. quasipneumoniae*; Table 1). Mapping of the presence or absence of the sequence region expected to be amplified by the primers was performed across *Klebsiella* phylogenetic diversity (Fig. 1). Regarding the targets proposed for the entire Kp complex, this *in silico* analysis revealed that only *khe* and *tyrB* (25, 26) presented both high sensitivity and high specificity. We further analyzed *in silico* the primers target sites for these two genes using primer blast. The results showed that *khe* primers would be expected to amplify the same region in *Raoultella* spp. (77 bp, with a sequence identity of the homologous region of <80%, and thus not visible on Fig. 1), as well as an additional region (348 bp) in these organisms. Furthermore, *tyrB* primers also appear to be able to amplify the target region in *K. aerogenes* and *Raoultella* spp., consistent with the distribution of the target region (Fig. 1). Regarding the targets that were proposed to be specific for Kp1, target KpI50233a (24) proved to be the most specific and sensitive one (although with ability to also amplify *K. aerogenes* isolates), whereas the ones proposed for Kp3 (23) appeared unspecific or to lack sensitivity in our *in silico* analysis (Fig. 1). Regarding the targeted chromosomal class A beta-lactamase genes *bla*_SHV_, *bla*_OKP_, and *bla*_LEN_ (28), there appeared to be a lack of specificity (Fig. 1), which is explained by the high degree of sequence identity between these *bla* genes, even though they represent distinct targets. To clarify their distribution, we mapped the expected amplicon sequences of *bla*_SHV_, *bla*_OKP_, and *bla*_LEN_ using a higher threshold (92%, defined based on observed sequence similarity within and between the three gene families) (see Fig. S1 in the supplemental material). Using this threshold, we observed a high degree of specificity of the three families for their respective phylogroup/species, as expected (32). However, it was evident that horizontal gene transfer events implicating the beta-lactamase genes have occurred. In particular, the *bla*_SHV_ gene was often observed in non-Kp1 phylogroups (particularly Kp2 and Kp4) and even in species outside the Kp complex, such as E. coli or *Salmonella* spp. This can be attributed to the presence of *bla*_SHV_ on plasmids and was previously described (21). The gene *bla*_LEN_ was also observed in a few Kp1 genomes. These observations clearly render the use of the chromosomal beta-lactamase gene targets unreliable as phylogroup/species identification markers (Fig. S1).

**TABLE 1:** Molecular target sequences previously proposed for K. pneumoniae identification^*a*^

Target	Annotation	Amplicon (bp)	Targeted group	Reference (PubMed ID)
16S-23S rRNA ITS	tDNA-Ala	130	Kp complex	18579248
16S-23S rRNA ITS	tDNA-Ala/23S rDNA	260	Kp complex	18579248
*khe*	Hemolysin	77	Kp complex	19644019
*tyrB*	Tyrosine aminotransferase	931	Kp complex	23357944
KpI50233a	Putative acyltransferase	484	Kp1	25261063
KP878	Transferase	878	Kp1	25886267
KP888	Phosphohydrolase	888	Kp1	25886267
*bla*_SHV_	Chromosomal SHV	995	Kp1	28139276
*celB*	Cellobiose-specific PTS family enzyme IIC component	180	Kp1	31456171
KV770	Phosphoglycerate mutase	449	Kp3/5	25886267
KV1000	Thiopurine *S*-methyltransferase	499	Kp3/5	25886267
KV1615	*N*-Acetyltransferase	438	Kp3/5	25886267
*bla*_LEN_	Chromosomal LEN	485	Kp3/5	28139276
*bla*_OKP_	Chromosomal OKP	348	Kp2/4	28139276
ZKIR	*zur-khe* intergenic region	78	Kp complex	This study

a ITS, internal transcribed spacer; PTS, phosphotransferase system; *bla*, beta-lactamase.

### ZKIR primer design, PCR assay development, and optimization.

The *tyrB* and *khe* genes were previously proposed as targets for the specific detection of Kp (25, 26). As shown above, *tyrB* and *khe* targets were not totally specific for the Kp complex. We therefore attempted to define primers within the coding sequence of these genes but external to the previously proposed target fragments. However, a high identity was observed with non-Kp complex species when blasting the entire coding sequence (data not shown). Consequently, Kp complex-specific primers could not be designed within the coding sequence of these two genes.

**FIG 2:**
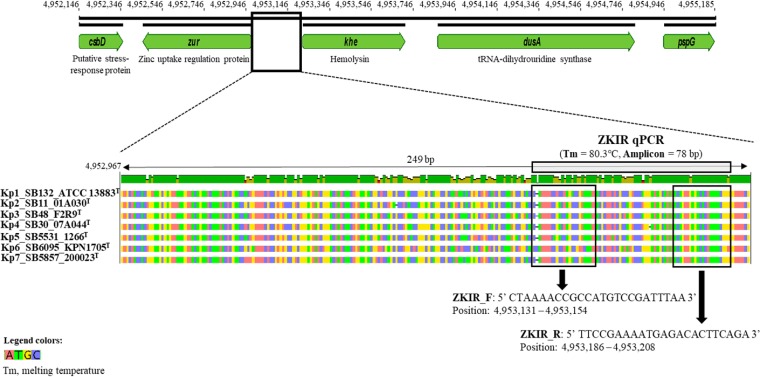
Genetic context of the ZKIR region on the genome of strain K. pneumoniae ATCC 13883^T^ (GenBank accession number GCA_000742135.1) and detailed location of ZKIR primers and amplicon within the 249-bp region that is specific for the K. pneumoniae species complex (boxed area).

Interestingly, investigation of sequences upstream of *khe* did reveal a sequence that was highly conserved within the Kp complex. This region located in the intergenic region (IR) upstream of *khe* is partly deleted in other species such as K. oxytoca and *Raoultella* spp. Since this 249-bp noncoding IR is located between *zur* (zinc uptake regulator) and *khe* (annotated as a putative hemolysin) genes (Fig. 2), it was named ZKIR for the *zur-khe* intergenic region.

A pair of primers (ZKIR_F and ZKIR_R, Fig. 2) targeting the ZKIR region was designed and tested *in silico*. When implemented in the SYBR green PCR assay using reference strain ATCC 700603 (known as K. pneumoniae, but belonging in fact to *K. quasipneumoniae* subsp. *similipneumoniae*), these primers successfully amplified a 78-bp sequence, with a melting temperature of 80.3°C. Sequencing of PCR products confirmed amplification of the target sequence.

The sensitivity and specificity of the primers were experimentally tested on 2 ng of purified DNA of representatives of Kp phylogroups Kp1 to Kp7 (Table 2) and non-Kp strains. The ZKIR_F and ZKIR_R primers amplified the target sequence in all tested isolates of the Kp complex, with a threshold cycle (*C_T_*) value ranging from 13 to 26 and melting temperatures of 80.1 to 80.7°C. In contrast, when the ZKIR PCR was performed on 88 non-Kp isolates, no amplification was observed, showing that the PCR did not yield false positives. Late, nonspecific amplification was recorded with a few isolates in the last cycles of the reaction (i.e., after cycle 35), and the melting temperatures were clearly lower or higher than 80°C, indicating a nonspecific amplification. All assays performed on non-Kp samples can therefore be considered negative.

**TABLE 2:** Bacterial strains tested to develop the ZKIR PCR assay^*a*^

Organism name (phylogroup)	Strain	Strain bank ID	Country	Isolation yr
*K. pneumoniae* (Kp1)	SB4-2	SB1067	Netherlands	2002
	ATCC 13883^T^	SB132	NA	NA
	MGH 78578	SB107	NA	1994
	None	SB1139	Netherlands	2002
	5-2	SB617	Netherlands	2000
	04A025	SB20	France	1997
	2-3	SB612	Netherlands	2000
	BJ1-GA	SB4496	France	2011
	NA	MIAE07651	France	2015
*K. quasipneumoniae* subsp. *quasipneumoniae* (Kp2)	01A030^T^	SB11	Austria	1997
	None	SB1124	Netherlands	2002
	U41	SB2110	Germany	1990
	10A442	SB224	Italy	1998
	99-1002	SB2478	Netherlands	1999
	18A451	SB255	Spain	1998
	11128	SB3445	NA	NA
	18A069	SB59	Spain	1997
	Kleb Ali 0320584	SB98	NA	NA
*K. variicola* subsp. *variicola* (Kp3)	01A065	SB1	Austria	1997
	07A058	SB31	Germany	1997
	IPEUC-1516	SB3278	France	1988
	CIP 53.24	SB3295	NA	NA
	Ørskov 1756/51	SB3301	NA	NA
	F2R9^T^	SB48	Mexico	NA
	6115	SB489	NA	NA
	Ørskov 4425/51	SB497	NA	NA
	Kp342	SB579	USA	NA
*K. quasipneumoniae* subsp. *similipneumoniae* (Kp4)	09A323	SB164	Greece	1997
	12A476	SB203	Netherlands	1998
	07A044^T^	SB30	Germany	1997
	325	SB3233	France	1975
	Ørskov 1303/50	SB3297	Turkey	NA
	Ørskov 4463/52	SB500	NA	NA
	CIP 110288	SB4697	China	2010
	1-1	SB610	Netherlands	2000
*K. variicola* subsp. *tropica* (Kp5)	Gal12	SB824	Mexico	NA
	CDC 4241-71	SB94	NA	NA
	885	SB5439	Madagascar	2016
	1266^T^	SB5531	Madagascar	2016
	1283	SB5544	Madagascar	2016
	1375	SB5610	Madagascar	2016
	814	SB5387	Madagascar	2015
“*K. quasivariicola*” (Kp6)	08A119	SB33	Germany	1997
	10982	SB6071	USA	2005
	01-467-2ECBU	SB6094	Madagascar	2015
	01-310A	SB6095	Madagascar	2013
	KPN1705^T^	SB6096	USA	2014
*K. africana* (Kp7)	200023^T^	SB5857	Senegal	2016
*K. michiganensis* (Ko1)	CIP 110787^T^	SB4934	USA	2010
	05A071	SB71	France	1997
	09A029	SB78	Greece	1997
*K. grimontii* (Ko6)	07A479	SB324	Germany	1998
	06D090	SB352	France	1998
	06D021^T^	SB73	France	1997
*K. oxytoca* (Ko2)	ATCC 13182^T^	SB175	NA	NA
	02A067	SB131	Belgium	1997
	NCTC 49131	SB136	NA	NA
*K. aerogenes*	None	MIAE07652	France	NA
	CIP 60.86^T^	SB3629	France	NA
	01A089	SB538	Austria	1997
	02A002	SB539	Belgium	1997
*R. terrigena*	ATCC 33257^T^	SB170	NA	NA
	17C143	SB313	Spain	1998
	V9813596	SB2796	Netherlands	1998
*R. planticola*	01A041	SB7	Austria	1997
	ATCC 33531^T^	SB174	NA	NA
	12C169	SB303	Netherlands	1998
*R. ornithinolytica*	ATCC 31898	SB171	NA	NA
*A. calcoaceticus*	ATCC 14987	None	USA	NA
*A. lwoffii*	None	MIAE07654	France	1998
*A. johnsonii*	ATCC 17909^T^	None	NA	NA
*A. pittii*	None	MIAE07655	France	1998
*A. caviae*	None	MIAE07656	France	NA
*A. hydrophila*	None	MIAE07657	France	NA
*A. tumefaciens*	C.58	MIAE07675	France	1996
*A. faecalis*	None	MIAE07658	France	NA
*B. cereus*	ATCC 53522	None	USA	NA
	None	MIAE07659	France	NA
*B. circulans*	None	MIAE07660	France	NA
*B. megaterium*	None	MIAE07661	France	NA
*B. subtilis*	None	MIAE07662	France	NA
*E. hoshinae*	DSM 13771^T^	None	France	NA
*E. tarda*	DSM 30052^T^	None	USA	NA
*E. hafnia*	None	MIAE07653	France	NA
*E. casseliflavus*	DSM 20680^T^	None	NA	1984
*E. faecalis*	DSM 12956	None	USA	NA
	DSM 20376	None	NA	NA
*E. faecium*	DSM 25389	None	Netherlands	NA
	DSM 6177	None	NA	NA
	DSM 25644	None	Georgia	NA
	DSM 25697	None	South Korea	NA
	DSM 13590	None	Germany	NA
	DSM 20477^T^	None	NA	NA
*E. gallinarum*	DSM 20628	None	NA	NA
*E. hirae*	DSM 20160^T^	None	NA	NA
*E. raffinosus*	DSM 5633^T^	None	USA	1979
*E. coli*	DSM 499	None	NA	NA
	None	MIAE02388	France	2015
	None	MIAE02376	France	2015
	None	MIAE02510	France	2015
	None	MIAE02381	France	2015
	None	MIAE02198	France	2015
*H. alvei*	DSM 30163^T^	None	NA	NA
*L. monocytogenes*	ATCC BAA-679 (EGDe)	None	NA	NA
	DSM 15675	None	NA	NA
	DSM 19094	None	United Kingdom	NA
*L. innocua*	None	MIAE07668	France	2018
*M. morganii*	None	MIAE07669	France	NA
*P. agglomerans*	DSM 3493^T^	None	Zimbabwe	1956
*P. mirabilis*	None	MIAE07670	France	NA
*P. vulgaris*	None	MIAE07671	France	NA
*P. rettgeri*	None	MIAE07672	France	NA
*P. stuartii*	None	MIAE07673	France	NA
*P. brassicacearum*	DSM 13227^T^	None	France	NA
*P. fluorescens*	DSM 50106	None	NA	NA
*P. gessardii*	CIP 105469^T^	None	France	NA
*P. jessenii*	CFBP4842	None	France	1995
*P. kilonensis*	DSM 13647^T^	None	Germany	NA
*P. libanenis*	DSM 17149^T^	None	Lebanon	1995
*P. lini*	DSM 16768^T^	None	France	NA
*P. monteilii*	DSM 14164^T^	None	France	1990
*P. putida*	ATCC 12633^T^	None	NA	NA
*P. rhodesiae*	DSM 14020^T^	None	France	NA
*P. thivervalensis*	CFBP5754	None	France	2000
*S. bongori*	DSM 13772^T^	None	NA	NA
*S. enterica*	DSM 554	None	NA	NA
*S. enteritidis*	None	Miae07674	France	2007
*S. subterranea*	DSM 16208^T^	None	USA	NA
*S. fonticola*	DSM 4576^T^	None	NA	NA
*S. liquefaciens*	DSM 4487^T^	None	Ireland	1997
*S. boydii*	DSM 7532^T^	None	India	NA
*S. aureus*	DSM 2569	None	South Korea	NA
*S. capitis*	DSM 20326^T^	None	NA	NA
*S. epidermidis*	DSM 20044^T^	None	NA	NA
*S. maltophilia*	DSM 50170^T^	None	USA	1961
*V. chagasii*	DSM 17138^T^	None	Norway	NA

a NA, information not available. A superscript “T” indicates a type strain. “SB” is an internal strain collection number of the Biodiversity and Epidemiology of Bacterial Pathogens unit, Institut Pasteur; MIAE, internal strain collection number of the UMR Agroecologie, INRA.

### Analytical sensitivity of the ZKIR PCR.

**FIG 3:**
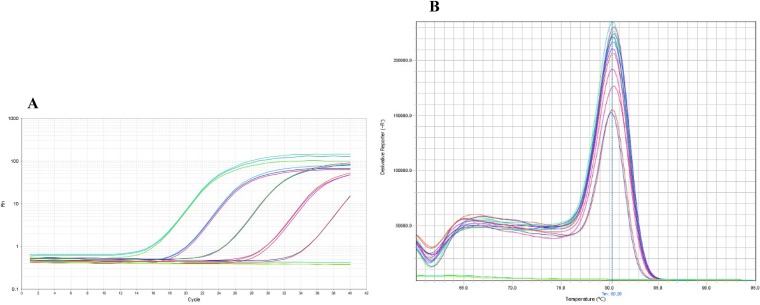
Amplification curves (A) and melting curve peaks (B) established using real-time PCR targeting the ZKIR region with serial dilutions of K. pneumoniae ATCC 13883^T^ DNA. Triplicate values with DNA concentrations of 7.5 ng, 750 pg, 75 pg, 7.5 pg, and 750 fg are presented, but results with lower dilutions are not.

**FIG 4:**
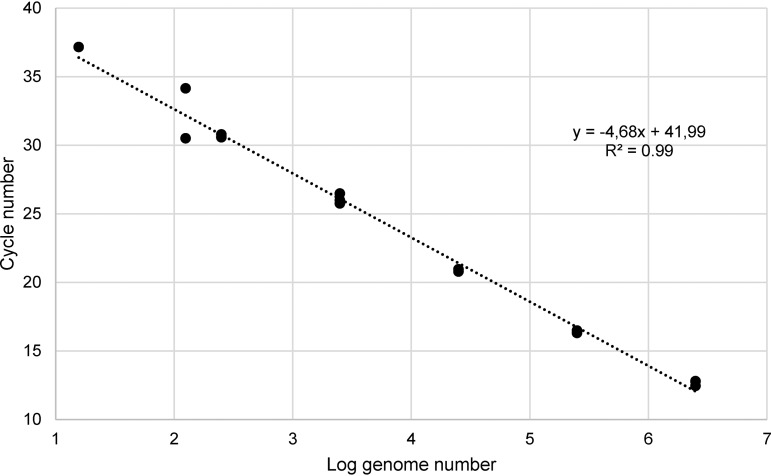
Standard curve established using real-time ZKIR PCR with serial dilutions of K. pneumoniae ATCC 13883^T^ DNA from 7.5 ng to 45 fg.

PCRs were performed with quantities of genomic DNA of K. pneumoniae ATCC 13883^T^ ranging from 7.5 ng (2.5 × 10^6^ genomes) to 15 fg (5 genomes) (Fig. 3A and B). The linearity of the assay was evaluated by plotting the *C_T_* values against the log_10_ calculated genome number (calculated genome size is about 3.0 fg of DNA per cell, based on a 5.54 Mb K. pneumoniae genome). *C_T_* values were closely proportional to the logarithm of the genome number (*R*^2^ = 0.99) (Fig. 4). The sensitivity of the assay was determined as 15 genomes (45 fg) per reaction mix. No amplification was observed at the lower DNA concentration of 15 fg corresponding to five genomes.

### Analytical sensitivity of the ZKIR assay on soil samples.

In order to assess the performance of the ZKIR assay for detection of Kp directly from soil samples, two soils (A and V) were spiked with bacterial concentrations ranging from 1.5 × 10^−1^ CFU/g to 1.5 × 10^4^ CFU/g. When soil samples were enriched in LB for 24 h and processed as described above, Kp was detected in all spiked microcosms except in two out of the three microcosms of soil A inoculated with the lowest Kp concentration. When the soil/LB suspension was tested prior to incubation, Kp was not detected in any of the spiked microcosms of soil V, whereas in soil A Kp could only be detected at the highest concentration (1.5 × 10^4^ CFU/g). Finally, when the ZKIR assay was performed using purified metagenomic DNA from soil, positive results were only observed in microcosms spiked with 1.5 × 10^4^ and 1.5 × 10^3^ CFU/g in both soils, whereas no positive signal was observed at lower concentrations. The enrichment step thus appeared critical to reach high sensitivity.

### Comparison of the ZKIR real-time PCR and culture-based methods for the detection of Kp in environmental samples.

After enrichment and ZKIR real-time PCR, Kp was detected in 41 of 96 (42.7%) assayed environmental samples, when the 1:10 dilution was used as template DNA (Table 3). When the 1:100 dilution was used, Kp was detected in a lower number of positive samples (38/96). The *C_T_* values were in the range 18.4 to 36.2. The 96 samples were processed in parallel with the culture-based method. Kp was not detected in any of the ZKIR-negative samples: when presumptive colonies were detected, they were always identified by matrix-assisted laser desorption ionization–time of flight mass spectroscopy (MALDI-TOF MS) as non-Kp and belonged to closely related species such as K. oxytoca, *Raoultella* spp., and *Serratia* spp. In contrast, Kp was isolated in 37 of the 41 ZKIR positive samples. These isolates were all identified as either K. pneumoniae or *K. variicola* by MALDI-TOF MS.

**TABLE 3:** Comparison between ZKIR qPCR and culture results obtained using samples collected in Auxonne, France, between July and September 2018

Source	Total no. of samples	No. (%) of samples	
		qPCR positive	Culture positive
Bulk soil	32	13 (40.6)	13 (40.6)
Roots	31	19 (61.3)	16 (51.6)
Leaves	29	8 (27.6)	7 (24.1)
Water	4	1 (25.0)	1 (25.0)
Total	96	41 (42.7)	37 (38.5)

Genomic characterization of 31 of these isolates (1 was contaminated, and 5 were not yet available) revealed a dominance of Kp1 (*n* = 20, 65%), followed by Kp3 (*n* = 10, 32%) and Kp4 (*n* = 1, 3%; see Table S1 in the supplemental material). Population diversity analysis based on multilocus sequence typing (MLST) (33) and core genome MLST (cgMLST) (24) revealed a high genotypic diversity, with 23 STs and 25 cgMLST types. In five cases, the strain detected in the soil was the same as the one present in the leaves and/or roots (e.g., SB6439 and SB6440; Table S1), showing colonization of several plant sites by the same strain. Only two isolates belonging to STs commonly found in the clinical settings were detected (ST37 and ST76). Interestingly, from the predicted O types, the O3 type represented 50% of Kp population, which contrasts with the nosocomial situation, where types O1 and O2 are dominant (2, 34), but not with human carriage, where the O3 type is also dominant (O3, 31%; O1, 19%; and O2, 17% [our unpublished results]). Also contrasting with the clinical epidemiology of Kp, a low level of antibiotic resistance and virulence genes was observed, with 93.5% of the strains presenting an ancestral “wild-type” susceptibility genotype (Table S1). The number of strains harboring plasmids detected in these environmental samples was also low (*n* = 9, 29%), as well as the number of plasmid-encoded heavy metal tolerance genes (mainly the silver and copper tolerance clusters). These results contrast with clinical and animal isolates, where plasmids and metal tolerance clusters are common (34; C. Rodrigues, unpublished results). As expected (1, 35), all Kp3 isolates harbored the *nif* cluster responsible for nitrogen fixation. Interestingly, the *nif* cluster was also present in one Kp1 isolate from soil (SB6181). The phylogenetic analysis (Fig. S2) of the *nif* cluster from our environmental isolates compared to a panel of reference strains (35) revealed that strain SB6181 (Kp1) branched within *K. variicola* strains (Kp3), showing that the *nif* cluster in this Kp1 strain was acquired via horizontal gene transfer from a *K. variicola* donor. Kp3 was also the inferred donor of the *nif* gene cluster for Kp5 and Kp6 *nif*-positive strains (Fig. S2).

## DISCUSSION

Routes of transmission of ecologically generalist human pathogens are usually complex and poorly understood. Proper risk management requires a holistic approach which has been theorized as the “One Health concept” (36). Despite the fact that the number of human infections caused by members of the Kp complex are on the rise and are increasingly resistant to antimicrobial treatment (1, 37), the ecology of Kp remains poorly understood. Identification of the various habitats in which Kp strives and the routes of transmission to humans, for example, through specific types of food, is critical in order to limit exposure. Large-scale sampling is needed to define the sources of Kp contamination, and such surveys will require sensitive, reliable, and cost-efficient detection methods.

Several molecular assays for the detection and/or identification of *Klebsiella* from clinical or food and environmental samples have been previously proposed. These methods allow detecting mainly K. pneumoniae (*sensu stricto*) and *K. variicola* (Fig. 1). However, the Kp complex currently encompasses seven taxa. Our *in silico* analyses showed that some previously described PCR assays could be useful for the identification of phylogenetic subsets of the Kp complex (Kp150233a for Kp1, *bla*_OKP_ for Kp2/Kp4, and *bla*_LEN_ for Kp3/Kp5).

Given that all members of the Kp complex can cause infections in humans or animals, developing a novel method with the ability to detect the Kp complex by targeting exhaustively all currently known Kp phylogroups would be an important advance. Although several previous targets were designed for the entire Kp complex, they predate recent taxonomic advances and have therefore not been validated on the entire phylogenetic breadth of this bacterial group. Here, we found by *in silico* approaches that *khe* and *tyrB* assays lack specificity, which may negatively affect Kp detection efforts especially when testing microbiologically complex samples from the environment, such as soil.

We therefore aimed to develop a novel real-time PCR assay and used an intergenic region located adjacent to the previously proposed target gene *khe*. Using well-defined reference strains representative of current taxonomy (20, 38, 39), we show that the novel ZKIR PCR real-time assay allows the accurate detection of all members of the Kp complex. We found complete specificity and sensitivity (i.e., phylogenetic coverage) of this assay based on our reference strains panel. To improve analytical sensitivity, a 24-h enrichment culture followed by an easy, fast, and cost-effective sample processing was implemented prior to molecular detection using the ZKIR assay. This was necessary for complex matrices such as soil or sewage, where microbial diversity and abundance are high, in which case direct detection of one particular group of organisms, which might be present only at low abundance, is challenging. This procedure turned out to be sensitive enough to detect a single Kp bacterium in 5 g of soil. The ZKIR assay also appeared slightly more sensitive than the SCAI (Simmons citrate agar with 1% inositol) culture-based method, which is based on the ability of *Klebsiella* strains to utilize citrate and inositol, itself previously shown to be highly sensitive and to recover most Kp members (40, 41). Therefore, this fast and easy novel molecular method represents a powerful approach for screening large numbers of samples. This will spare the time-consuming handling of numerous presumptive colonies necessary for confirmation of their identification, given that K. oxytoca, *Raoultella*, *Serratia*, and *Enterobacter* are able to form colonies on SCAI agar, with morphological characteristics similar to Kp (40, 42). As such, the ZKIR protocol was significantly faster than culture, since results were available on average 26 h after sampling (24-h enrichment, sample treatment, and PCR), while up to 96 h were necessary when using the culture-based method (24-h enrichment, plate incubation, colony purification, and MALDI-TOF identification).

Interestingly, the implementation of the ZKIR PCR real-time assay evidenced a high (43%) detection rate of Kp from environmental samples, which represent niches that are still underexplored for Kp presence and biological characteristics. The high detection rate was largely confirmed by culture, suggesting a low rate of false positive qPCR results. This novel and original sampling strategy allowed us to explore the genomic features of environmental Kp populations. A strong contrast with Kp isolates typically recovered in the clinical setting was observed. First, a high genetic diversity was observed, with no predominant sublineage. This situation contrasts with MDR or hypervirulent Kp populations, from which frequent sublineages (so-called high-risk clones) are recovered. Second, environmental Kp isolates were almost devoid of antibiotic resistance and virulence genes, in conspicuous contrast with clinical samples (43). Finally, the genomic characteristics of environmental Kp isolates revealed interesting biological features, such as a frequent O3 O-antigen type and the horizontal transfer of nitrogen fixation gene cluster into Kp1, the main phylogroup associated with human infections. These data call for further studies into the biology of soil Kp isolates, which may reveal interesting novel adaptive strategies of this important generalist pathogen. Finally, our findings suggest that environmental Kp populations differ from clinical Kp populations, which implies indirect and possibly complex epidemiological links between environmental and clinical Kp. The ZKIR PCR real-time assay developed here is expected to enable future large-scale studies into this important question.

In conclusion, the ZKIR assay is a new tool for Kp detection that is highly specific, sensitive, reliable, and cost effective. To accelerate uptake of this method, the corresponding protocol was released publicly on protocols.io (https://doi.org/10.17504/protocols.io.7n6hmhe). This simple method can easily be implemented in laboratories equipped with real-time PCR thermocyclers (MedVetKlebs consortium, unpublished results). Using the ZKIR method after a short culture enrichment step greatly enhances sensitivity. As this rapid screen of samples allows one to focus only on ZKIR-positive samples for more labor-intensive downstream microbiological isolation and characterization, it is our hope that it will contribute to advance knowledge on the biology of environmental Kp and on the reservoirs and transmission routes of this increasingly important group of pathogens.

## MATERIALS AND METHODS

### Bacterial strains and culture conditions.

A panel of 136 strains from the collections of the Institut Pasteur (Paris, France) and INRA (Dijon, France) was used (Table 2). It included the K. pneumoniae type strain ATCC 13883^T^ and 47 other strains of the Kp complex (the Kp1 to Kp7 phylogroups, including type strains) isolated from patients, animals and outdoor environments from multiple geographic locations. A total of 88 non-Kp bacterial strains from other species of the genus *Klebsiella*, closely related genera (*Raoultella* and *Enterobacter*), and other species that have similar environmental niches were included for comparison. All strains were regenerated by streaking on tryptone soy agar (TSA; Conda, Spain). After 24 h at 37°C, single colonies were transferred to 10 ml of tryptone soy broth (TSB; Conda). Bacterial suspensions were collected after 24 h of incubation at 37°C for further experiments.

### Bacterial DNA extraction.

The bacterial DNA was extracted according to a homemade protocol. One milliliter of the 24-h bacterial culture was centrifuged (7,000 × *g*, 10 min, 4°C), and the pellet washed with sterile water and suspended in 800 μl of Tris (50 mM, pH 8) in which 10 μl of EDTA (500 mM, pH 8), 115 μl of lysozyme (10 mg/ml, incubation at 37°C for 30 min), 57 μl of 20% sodium dodecyl sulfate (incubation at 65°C for 15 min), 5 μl of RNase (10 mg/ml, incubation at 37°C for 30 min), and 23 μl of proteinase K (6 mg/ml, incubation at 37°C for 1 h and 30 min) were added. Finally, a 1:1 volume of phenol-chloroform-isoamyl alcohol (25/24/1) was added. The suspension was shaken 3 min at 15 rpm and then centrifuged 3 min at 20,000 × *g*. The supernatant was transferred in a new tube, and a 1:1 volume of chloroform/isoamyl alcohol (24/1) was added. After shaking (3 min, 25 rpm) and centrifugation (3 min, 20,000 × *g*), this step was repeated once. After transfer of the supernatant in a new tube, a 1:10 volume of NaCl (5 M) was added (shaking for 1 min at 15 rpm). Finally, a 1:1 volume of isopropanol (shaking for 3 min at 15 rpm) was added. Precipitated DNA filaments were collected with a glass Pasteur pipette and transferred in 400 μl frozen ethanol (70%). After shaking for 3 min at 15 rpm, DNA filaments were collected, dried at room temperature, and finally dissolved in 100 μl of TE (pH 8). The DNA concentration was estimated using a NanoDrop 2000 spectrophotometer (Thermo Fisher Scientific) and adjusted at 20 ng/μl. Purified DNA was stored at –20°C.

A simpler protocol was implemented for the PCR specificity assay and validation study. Bacteria grown in TSB or enrichment medium were centrifuged 5 min at 13,000 × *g*. Pellets were suspended in 200 μl of Tris-HCl (5 mM, pH 8.2) supplemented with 13 μl of proteinase K (1 mg/ml). After 2 h of incubation at 55°C, proteinase K was inactivated during 10 min of boiling. The cell debris were discarded by centrifugation (5 min at 13,000 × *g*), and the supernatant was stored at – 20°C before use for real-time PCR.

### Soil DNA extraction.

Soil metagenomic DNA was extracted from 2-g aliquots of soil using the modified ISO standard 11063 method (44). Crude soil DNA extracts were first purified onto PVPP (polyvinylpolypyrrolidone) minicolumns (Bio-Rad, France) and then with the GeneClean Turbo kit (MP Biomedicals, France) according to the manufacturer’s protocol (44). Purified DNA concentrations were determined using a NanoDrop 2000 spectrophotometer.

### *In silico* identification of the target DNA region.

The phylogenetic distribution of molecular targets that were previously described in the literature for Kp detection, was investigated *in silico*. All available Kp genomes were downloaded from the NCBI database (*n* = 3,552, February 2018) and combined with the ones of Institut Pasteur’s internal reference collection (*n* = 670) (20, 38, 39), representing a total of 4,222 genomes, which included *Klebsiella* species and closely related *Raoultella* species. The average nucleotide identity (ANI) metric was used to classify the Kp genomes in each of the phylogroups. To avoid redundancy in the data set and due to the difficulty of analyzing phylogenetically 4,222 genomes, in the case of the genomes belonging to the Kp complex (which represented the majority of the data set), we selected unique representatives of each MLST sequence type (ST), as defined using the *mlst* software tool (https://github.com/tseemann/mlst). This resulted in a final collection of 1,001 genomes that were tested for the presence of targets from the literature. Nucleotide BLAST (BLASTN) was used to detect the presence of the target sequences in the 1,001 genomes, with 80% nucleotide identity (also with 92% in the case of *bla*_SHV_, *bla*_OKP_, and *bla*_LEN_ targets) and 80% length coverage as cutoffs. Primer-BLAST (https://www.ncbi.nlm.nih.gov/tools/primer-blast/) was used (with default parameters) to check the distribution of primer target sites in the genomic sequences. Specificity was defined as the proportion of target organisms among those expected to be amplified by the target assay. Sensitivity was defined as the proportion of target organisms expected to be amplified by the target assay. A phylogenetic tree was constructed (45), and the output was visualized using iTOL (https://itol.embl.de/; Fig. 1; see also Fig. S1 in the supplemental material).

### Design of primers.

The Kp *tyrB* and *khe* gene sequences (accession numbers: AF074934.1 and AF293352.1) were aligned against the genome sequences of K. pneumoniae (CP012744.1, CP012743.1, and CP028787.1), *K. variicola* (CP008700.1, CP013985.1, and CP017289.1), *K. quasipneumoniae* (CP014071.1, CP023478.1, and CP029432.1), and *K. quasivariicola* (CP022823.1) using BLASTN to identify conserved genomic regions within the Kp complex. Sequences of closely related bacterial species K. oxytoca (CP027426.1), Raoultella ornithinolytica (CP010557.1), *R. planticola* (CP026047.1), and *K. aerogenes* (CP014029.2) were added in the alignments to confirm the specificity of these regions for the Kp complex.

Primer sets were designed using Primer3Plus (http://www.bioinformatics.nl/cgi-bin/primer3plus/primer3plus.cgi). The specificity of the predicted primer sets and amplicons was checked by applying BLASTN on the GenBank nucleotide collection (nr/nt) from the NCBI database, and Multiple Primer Analyzer (Thermo Fisher) was run to check for dimer formation. The best candidate primer sets defined by this *in silico* approach were ordered for synthesis at Eurogentec; among these was the primer pair ZKIR_F (5′-CTA-AAA-CCG-CCA-TGT-CCG-ATT-TAA-3′) and ZKIR_R (5′-TTC-CGA-AAA-TGA-GAC-ACT-TCA-GA–3′).

### Real-time PCR.

All real-time PCR assays were performed on an ABI StepOne real-time thermocycler (Fisher Scientific, France) with the following temperature program: 95°C for 3 min and 40 cycles at 95°C 10 s and 60°C for 1 min. Melting curves were generated with temperature increments of 0.3°C per cycle from 60 to 95°C. DNA was amplified in a 20-μl PCR mix containing 10 μl of Takyon Low Rox SYBR MasterMix dTTP Blue (Eurogentec, Belgium), 2 μl of each primer (final concentrations, 300 nM), 2.5 μl of template DNA, and 3.5 μl of PCR-grade water. For the detection of Kp from environmental samples, 0.5 μl of T4 gene 32 protein (Sigma-Aldrich) was added to the PCR mix. The specificity and cross-reactivity of the ZKIR assay was evaluated with 2 ng of purified DNA of 48 Kp complex strains and 88 non-Kp isolates (Table 2).

Two universal primers targeting a 174-bp region of the 16S rRNA gene of *Eubacteria*, 341f (5′-CCT-ACG-GGA-GGC-AGC-AG-3′) and 515r (5′-ATT-CCG-CGG-CTG-GCA-3′), were used for positive-control PCRs, as described in a previous report (46).

### Standard curve development and sensitivity assessment.

Aliquots (2.5 μl) adjusted to 7.5 ng, 750 pg, 75 pg, 7.5 pg, 750 fg, 375 fg, 45 fg, and 15 fg of genomic DNA of K. pneumoniae ATCC 13883^T^ were prepared in triplicate and amplified using the optimized PCR conditions described above. The results were analyzed with the StepOne data analysis software, and the PCR efficiency was determined. The genome number expressed as a logarithm was plotted against *C_T_* values, and the correlation coefficient (*R*^2^) of the standard curve was calculated.

### Comparison of the ZKIR PCR and culture methods for the detection of Kp in environmental samples.

Ninety-six environmental samples collected between July and September 2018 (23 in July, 39 in August, and 34 in September) in Auxonne (Burgundy, France) were analyzed for Kp presence using the ZKIR assay and culture methods in parallel. Samples corresponded to bulk soils (*n* = 32), roots (*n* = 44), leaves (*n* = 29), and irrigation water (*n* = 4) and were processed in the lab within 24 h after sampling. Then, 10-g samples of soil were weighed in 180-ml pots (Dutscher, France). Plant leaves and roots were properly cut, cleaned with sterile water, and transferred in 180-ml pots. Processed samples were suspended in 90 ml of lysogeny broth (LB; 5 g of NaCl, 5 g of yeast extract, and 10 g of tryptone for a 1-liter final volume) supplemented with ampicillin (10 mg/liter, ampicillin sodium salt; Sigma-Aldrich). Water samples (500 ml) were filtered through a 0.25-μm-pore-size membrane (Millipore, France). The membrane was incubated in 20 ml of LB supplemented with ampicillin as described above. After 24 h of incubation at 30°C, enrichments were vortexed, and 500-μl aliquots were centrifuged (5 min at 5,800 × *g*) and washed with sterile water. The pellet was suspended in 500 μl of sterile water and boiled for 10 min. Boiled enrichments were 10-fold diluted (1:10 and 1:100), and the dilutions were used as templates for real-time PCR.

In parallel, enrichments were serially diluted (1:10 to 1:10,000) in sterile water before plating on Simmons citrate agar enriched with 1% inositol (SCAI medium) (40). Plates were incubated 48 h at 37°C. Each plate was screened for presumptive colonies of *Klebsiella* (large, yellow, dome-shaped colonies), and ten candidate colonies were purified on SCAI medium and identified using MALDI-TOF MS (MALDI Biotyper; Bruker) according to the MALDI Biotyper Compass database, version 4.1.80 (Bruker Daltonics, Germany). In addition, whole-genome sequencing was performed for environmental Kp isolates (*n* = 31) using the NextSeq-500 sequencing platform (Nextera XT library; 2 × 150 nucleotides). Genomic assemblies were obtained using SPAdes v3.9. MLST and cgMLST were performed using the BIGSdb-Kp Web tool (https://bigsdb.pasteur.fr/klebsiella/klebsiella.html). This resource coupled with Kleborate (https://github.com/katholt/Kleborate) was used to search for antibiotic resistance, virulence, and heavy metal tolerance genes and to predict capsular types. PlasmidFinder was used to look for plasmid replicons (https://cge.cbs.dtu.dk/services/PlasmidFinder/). To construct the tree based on *nif* cluster genes (see Fig. S2 in the supplemental material), the DNA region between the *nifQ* and *nifJ* genes was extracted from the *nif* carrying strains and from a set of reference strains carrying this cluster (35). Sequences were aligned with MUSCLE, and IQ-TREE (http://iqtree.cibiv.univie.ac.at) was used to reconstruct the maximum likelihood phylogeny using the HKY+I+F+G4 model.

### Determination of the limit of detection of the ZKIR assay in artificially spiked soils.

Two soils with contrasting edaphic characteristics (sandy soil A and clay soil V) and free of indigenous Kp, according to the procedure described above, were used. A bacterial suspension of K. pneumoniae ATCC 13883^T^ was serially diluted in sterile water and dilutions were enumerated on TSA plates. Then, 5-g aliquots of each soil were spiked with these decreasing Kp dilutions, resulting in final concentrations from 1.5 × 10^4^ to 1.5 × 10^−1^ CFU/g. Soil samples were prepared in triplicate with three independently grown inoculums at four dilutions (36 spiked microcosms for each soil). Negative controls were prepared by adding the same volume of sterile water (three microcosms for each soil). All 78 spiked and control soil microcosms were enriched for 24 h at 30°C in 45 ml of LB supplemented with ampicillin (10 mg/liter; Sigma-Aldrich). Next, 500-μl aliquots of enrichment broth were sampled before and after the 24-h enrichment step, centrifuged 5 min at 5,800 × *g*, and washed with sterile water. The pellet was suspended in 500 μl of sterile water and boiled for 10 min. Boiled enrichments were serially 10-fold diluted (1:10 to 1:100,000), and the diluted suspensions were used for real-time PCR. Moreover, metagenomic DNA was extracted from spiked soils as described above under “Soil DNA extraction.”

### Data availability.

The detailed ZKIR qPCR operating procedure was made publicly accessible to the scientific community through the protocols.io platform (https://doi.org/10.17504/protocols.io.7n6hmhe). Genomic sequences generated in this study were submitted to the European Nucleotide Archive and are accessible under the BioProject number PRJEB34643.

## Supplementary Material

Supplemental file 1

Supplemental file 2
