# Supplementary material for: The ZKIR Assay, a Real-Time PCR Method for the Detection of Klebsiella pneumoniae and Closely Related Species in Environmental Samples
Source: Appl Environ Microbiol. 2020 Mar 18;86(7):e02711-19. doi: 10.1128/AEM.02711-19 (PMC7082575; doi:10.1128/AEM.02711-19)
Supplement: Supplemental file 1 [file AEM.02711-19-s0001.pdf]

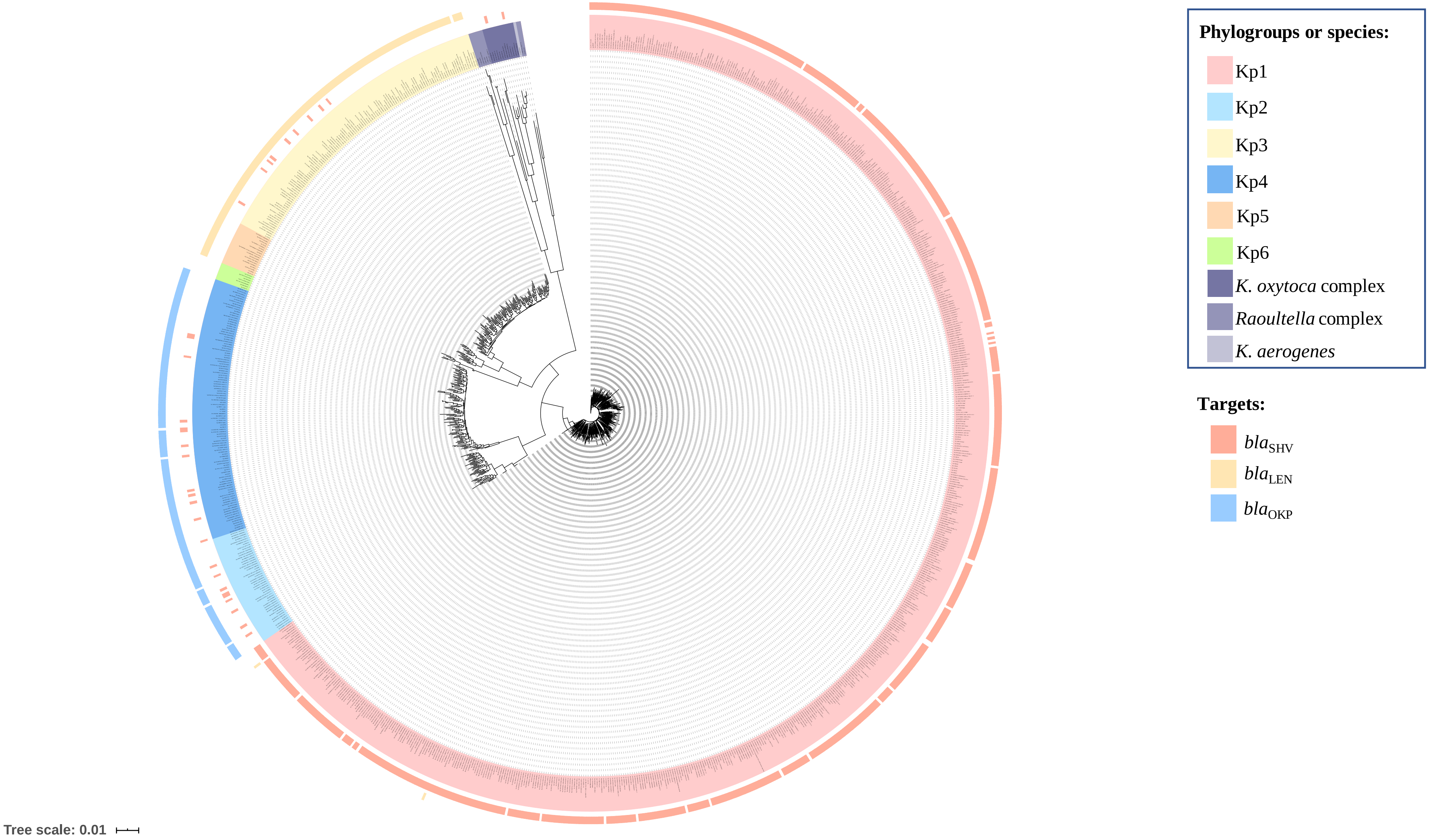

Figure S1. Phylogenetic distribution of the *bla*$_{SHV}$, *bla*$_{LEN}$ and *bla*$_{OKP}$ PCR targets (expected amplicons) in *K. pneumoniae*. The target details are given in Table 2. The inner circle colored sectors correspond to *K. pneumoniae* phylogroups or other *Klebsiella* species (see color key). The three external circles show the distribution of the target sequences, as detected in the genomic sequences using BLASTN with 92% nucleotide identity and 80% length coverage.

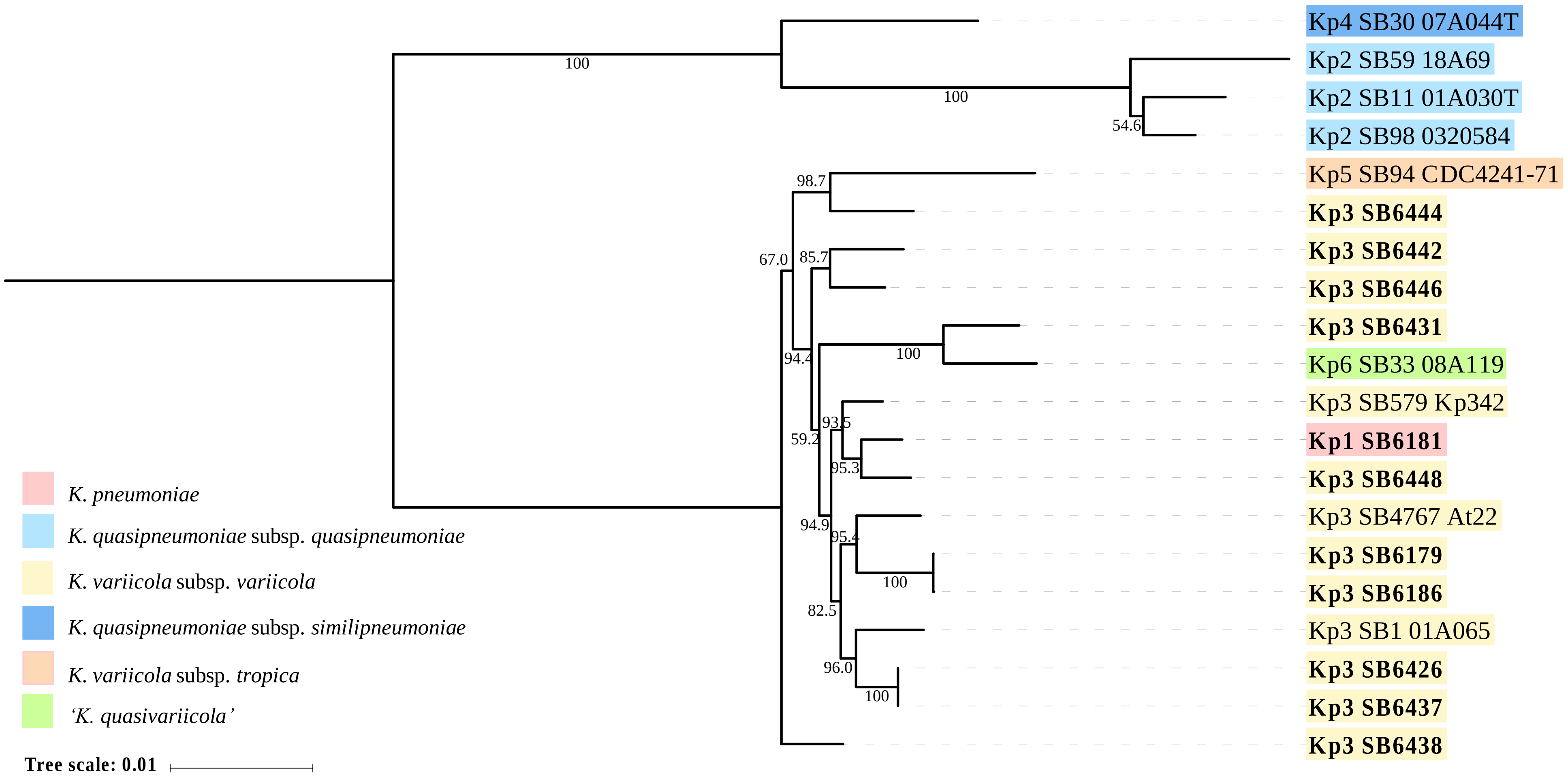

FIGURE S2. Maximum likelihood phylogeny based on the *nif* cluster gene sequences using the HKY+I+F+G4 model. The scale bar indicates the number of nucleotide substitutions per site. Node labels indicate bootstrap values. The isolates from this study are represented in bold, whereas other isolates are references taken from Blin *et al*. (Environmental Microbiology 2017 19:1881-1898).
